# COVID-19 impact on retinal detachment in Germany: seasonal peaks persist and shifts in surgical trends

**DOI:** 10.1007/s10792-025-03706-z

**Published:** 2025-09-01

**Authors:** Ahmad Samir Alfaar, Efstathios Vounotrypidis, Armin Wolf

**Affiliations:** 1 Ophthalmology Department, Ulm University Hospital, Ulm, Germany; 2 Medical Neuroscience Program, Charité–Universitätsmedizin Berlin, Berlin, Germany; 3 St. Paul Eye Unit, Royal Liverpool University Hospital, Liverpool, UK

**Keywords:** COVID-19, Retinal detachment, Rhegmatogenous retinal detachment, Tractional retinal detachment, Seasonality, Surgical trends, Vitrectomy, Reoperation rates, Healthcare disruption, Germany

## Abstract

**Purpose:**

This study investigated how the COVID-19 pandemic coincided with changes in incidence, seasonality, and surgical management of rhegmatogenous retinal detachment (RRD) and tractional retinal detachment (TRD) in Germany.

**Methods:**

We performed an observational, retrospective analysis using data from the Diagnosis-Related Group database, covering 116,386 retinal detachment admissions between January 2019 and December 2022. RRD (ICD-10 H33.0) and TRD (ICD-10 H33.4) cases were examined separately. Seasonal decomposition and correlation analyses were applied to identify trends in admission numbers, surgical choices, and reoperations before and after the onset of COVID-19.

**Results:**

A total of 96,620 RRD and 19,766 TRD admissions were analyzed. Post-pandemic, RRD decreased by 8.5%, whereas TRD declined by 2.9%. Despite disruptions, established seasonal peaks remained evident, with RRD peaking in July and TRD in March and August. A negative correlation between COVID-19 deaths and RRD cases suggested a temporal association that may reflect barriers to timely care during pandemic peaks. Surgical practice shifted toward more definitive vitrectomy approaches, and reoperation rates for RRD rose slightly from 12.1 to 12.9%.

**Conclusion:**

The pandemic correlated with reduced RRD admissions, smaller declines in TRD, and persistent seasonality in Germany. These findings underscore the importance of adaptive healthcare strategies that preserve timely surgical interventions and resource allocation for sight-threatening conditions during public health crises.

**Supplementary Information:**

The online version contains supplementary material available at 10.1007/s10792-025-03706-z.

## Introduction

Retinal detachment (RD) represents a critical ophthalmological emergency requiring immediate surgical intervention to mitigate the risk of irreversible vision loss. Over the years, surgical advancements have improved patient outcomes, but RD continues to impose a significant burden on healthcare systems, especially with increasing incidence over years [1, 2]. 

In 2020, the global healthcare landscape was dramatically altered by the emergence of the COVID-19 pandemic. The pandemic has had wide-reaching impacts on healthcare systems worldwide, including the postponement or cancellation of elective surgeries. Among these are ophthalmological procedures, potentially leading to delayed care and complications [3, 4]. While numerous studies have explored the immediate repercussions of the pandemic on healthcare delivery, scant research exists on its specific impact on conditions like RD that rely on timely medical intervention for optimal outcomes, and cover the whole crisis period [5, 6]. Moreover, the intersection of RD's urgency with COVID-19-related healthcare disruptions poses unique challenges. Delays in surgical intervention for RD are associated with worse outcomes, increased reoperation rates, and changes in the choice of surgical procedures.

This study aims to investigate the impact of the COVID-19 pandemic on RD cases in Germany, with specific hypotheses that rhegmatogenous retinal detachment (RRD) admissions would show a larger relative decline and disruption of seasonal peaks compared to TRD, and that changes may vary across age groups and surgical modalities.

## Methods

### Study design

This is an observational, retrospective study using secondary data to investigate the impact of the COVID-19 pandemic on retinal RD cases in Germany from January 2019 to December 2022.

### Setting

The study focuses on Germany, including a variety of healthcare settings ranging from community hospitals to tertiary care centers. The analysis includes data from both nonprofit and public hospitals.

### Data source and retrieval

Data for this study were extracted from the Diagnosis-Related Group (DRG) statistics database, as mandated by the Hospital Remuneration Act (KHEntgG, Sect.  21) [7]. The data were retrieved using the G-DRG browser managed by the Institute for the Remuneration System in Hospitals (InEK), accessed on 16.09.2023 [8]. The dataset includes records of patients admitted with a diagnosis of retinal detachment, identified by the ICD-10 codes H33.0 (Rhegmatogenous RD) and H33.4 (Tractional RD), from January 1, 2019, to December 31, 2022. In Germany, retinal detachment surgeries are conducted during hospital admissions, making this hospital-driven dataset particularly suitable for analyzing incidence and treatment dynamics. This contrasts with practices such as in the USA, where some surgeries may be performed as office procedures, and therefore hospital admission data might not fully capture all instances of the surgery [9]. Patients were categorized into age groups as follows: 'PreSchool' (ages 0–5 years), 'School' (ages 6–17 years), 'Early Work' (ages 18–39 years), 'Late Work' (ages 40–64 years), and 'Retirement/Post-Work' (ages 65 years and older).

### Participants and variables

The study population comprises patients who underwent surgical intervention for RD. Patients with unknown admission dates were excluded from the study. The primary outcome variables include the monthly incidence of RD cases and COVID-19 deaths. COVID19 deaths were used as a proxy indicator for the severity of the pandemic, and were extracted from the Robert Koch Institute website (www.rki.de). Secondary variables involve age groups, reoperation rates (Code 5-983), choice of surgical procedures, and hospital categories.

### Statistical methods

Descriptive statistics were computed for all variables, including central tendency and dispersion measures. Statistical tests, including F-tests, were conducted to compare the slopes of regression lines fitted to the data for each variable across the two periods. Seasonal decomposition of time series (STL) was applied to examine the seasonality in RD cases and other variables. Stationarity of all time series was assessed using the Augmented Dickey-Fuller (ADF) test [10]. If non-stationarity was detected at *p* < 0.05, the series was differenced once before further analysis including Granger causality testing. The strength of seasonality was calculated as the ratio of the variance of the seasonal component to the variance of the original time series, in accordance with Hyndman and Athanasopoulos (2018) [11]. Pearson's correlation coefficients were calculated to investigate relationships between incidence, COVID-19 deaths, and other secondary variables in RRD. A time-lagged correlation analysis was performed up to a 12-month lag to explore the delayed effects of COVID-19 deaths on RRD cases. Sensitivity analyses were conducted to assess the robustness of our findings, especially concerning potential biases. For inferential statistics, Huber regression was implemented with a tuning constant *c* = 1.345, which provides a balance between statistical efficiency and robustness [12]. The loss function used penalized large residuals linearly and smaller ones quadratically, as per standard implementation. For all statistical tests, a p-value of less than 0.05 was considered statistically significant.

All tests were two-tailed. All statistical analyses were performed using Python's Pandas for data manipulation, SciPy and scikit-learn for statistical modeling, and Matplotlib for data visualization. Some figures were plotted using Tableau Version 2024.3.

### Ethical considerations

The study adheres to ethical guidelines. The data were provided as anonymized statistics to the investigators.

## Results

### Study population

Between January 2019 and December 2022, a total of 116,386 RD admissions were analyzed, comprising 96,620 cases of RRD and 19,766 cases of TRD. The cohort demonstrated a male predominance, with males accounting for 64.5% of RRD cases and 62.0% of TRD cases. The mean hospital admission duration across all RD cases was 3.16 days (standard deviation [SD] 1.63 days), with the majority of admissions being of usual duration (90.9%).

In RRD cases, the 'Late Work' age group (40–64 years) constituted the largest proportion of patients at 51.8%, followed by the 'retirement' age group (≥ 65 years) at 42.8%. The 'Early Work' (18–39 years), 'School' (6–17 years), and 'Pre-School' (0–5 years) age groups accounted for 4.7%, 0.6%, and 0.1% of cases, respectively. TRD cases had a similar age distribution, with 50.9% in the 'Late Work' group and 47.3% in the 'retirement' group (Table 1).

**Table 1:** Basic characteristics of Rhegmatogenous and Tractional Retinal Detachment patients

	H33.0 - Rhegmatogenous Retinal Detachment				H33.4 - Tractional Retinal Detachment			
	2019	2020	2021	2022	2019	2020	2021	2022
N	23,885	22,225	23,942	26,568	5045	4915	4855	4951
Male %	64.3%	64.6%	64.4%	64.7%	61.4%	62.4%	61.5%	62.1%
Male N	15,368	14,355	15,422	17,178	3100	3068	2985	3077
Female %	35.7%	35.4%	35.6%	35.3%	38.6%	37.5%	38.5%	37.8%
Female N	8516	7870	8519	9389	1945	1845	1870	1873
Mean (SD) Admission Time	3.33 (1.68)	3.22 (1.75)	3.11 (1.58)	3.01 (1.55)	3.33 (1.77)	3.24 (2.00)	3.08 (1.83)	3.04 (1.72)
PCCL 0	93.0%	92.7%	92.5%	90.9%	80.0%	78.1%	78.4%	77.3%
PCCL 1	4.0%	4.5%	4.8%	6.3%	14.7%	16.2%	15.9%	17.6%
PCCL 2–6	3.0%	2.7%	2.7%	2.8%	5.2%	5.7%	5.7%	5.2%
Age 0–5 Years	0.1%	0.1%	0.1%	0.1%	0.3%	0.3%	0.5%	0.4%
Age 6–17	0.6%	0.5%	0.6%	0.5%	1.8%	1.3%	1.3%	1.6%
Age 18–39	4.9%	4.8%	5.0%	4.3%	8.9%	8.5%	9.0%	9.1%
Age 40–64	52.3%	52.8%	51.2%	51.2%	41.5%	41.4%	42.8%	42.3%
Age 65 +	42.2%	41.8%	43.1%	43.9%	47.6%	48.4%	46.4%	46.6%

Pars plana vitrectomy was the predominant surgical intervention for RRD, utilized in approximately 84% of cases. Reoperations were required in 12.8% of RRD cases. Regarding hospital characteristics, 52.8% of RD cases were managed in large hospitals with 1,000 beds or more. Public hospitals handled the majority of cases (71.1%), followed by nonprofit hospitals (15.6%).

### Seasonality of RD admissions

**Fig. 1:**
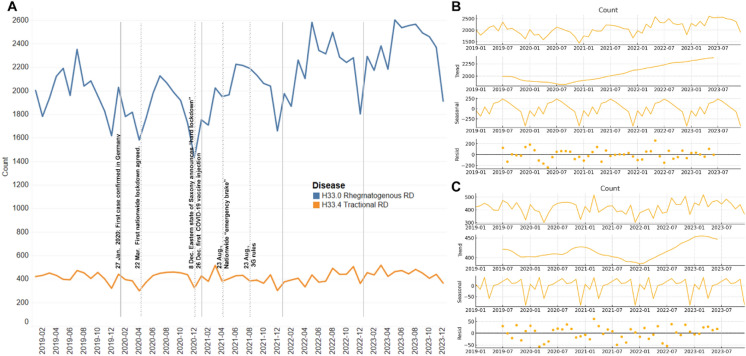
Time series Decomposition of number of admissions related to retinal detachment surgeries. A. Raw data for both Rhegmatogenous and Tractional Retinal Detachment with relation to major COVID19 Events in Germany. B. Seasonal variations and trends in Rhegmatogenous Retinal Detachment. C. Seasonal variations and trends in Tractional Retinal Detachment

Time-series analysis revealed significant seasonal patterns in RD admissions. For RRD, admissions consistently peaked during the summer months, particularly in July, accounting for approximately 56.6% of the total variance in RRD cases (Fig. 1B, Supplementary Fig. 1). This seasonal pattern persisted throughout the study period, although the peak shifted to May in 2022. TRD admissions exhibited peaks in March and August, contributing to 18.7% of the annual variance (Fig. 1C).

A moderate positive correlation was observed between monthly admissions of RRD and TRD (*R* = 0.59, *p* < 0.01), suggesting that external factors such as healthcare access and patient behavior may simultaneously influence both types of RD.

### Impact of COVID-19 on RD admissions

**Fig. 2:**
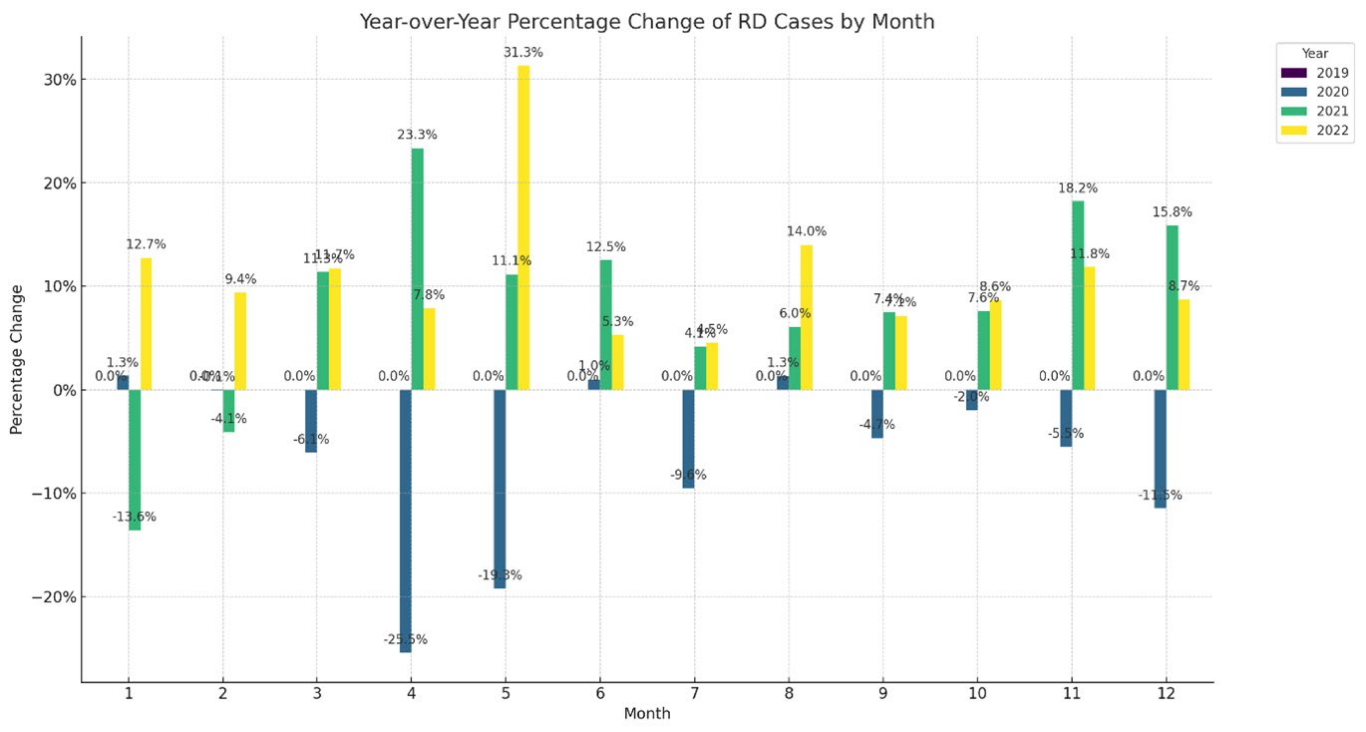
Change of admissions compared to 2019 (Rhegmatogenous Retinal Detachment)

Comparing the 12-month periods before and after the onset of the COVID-19 pandemic (lockdown initiated in March 2020), there was a significant decrease in RRD admissions. Total RRD cases declined by 8.5%, from 23,910 pre-pandemic to 21,876 post-pandemic (*p* = 0.048), with the decline most pronounced during mid-year months (Fig. 2, Supplementary Table 1, Supplementary Fig. 1). In contrast, TRD admissions experienced a smaller decline of 2.9% post-pandemic that recovered rapidly, indicating that TRD was less correlated with pandemic-related disruptions.

Analysis of age group fluctuations revealed minimal changes within 2%. There was a slight increase in RRD admissions among the 'Early Work' age group towards the end of the first wave and peak of the second wave (March 2021), accompanied by an increase in the 'retirement' age group in March 2022 and a decrease in the 'Late Work' age group. These fluctuations may reflect variations in exposure risk or healthcare-seeking behavior among different age groups during the pandemic. Notably, the 'Post-Work' age group was slightly more affected during the pandemic, as indicated by a decrease in admissions (Supplementary Fig. 2).

The gender distribution remained stable for RRD, with males constituting approximately 65% of cases in both periods.

A significant negative correlation was found between monthly RRD admissions and COVID-19 deaths (R^2^ = 0.40, *p* = 0.001; correlation coefficient = − 0.63), suggesting that higher COVID-19 mortality rates were associated with fewer RRD admissions (Fig. 4). This inverse correlation suggests that pandemic severity may have influenced patients' willingness or ability to seek timely ophthalmic care or a decline in daily activities that may result in retinal detachment.

### Changes in surgical management

**Fig. 3:**
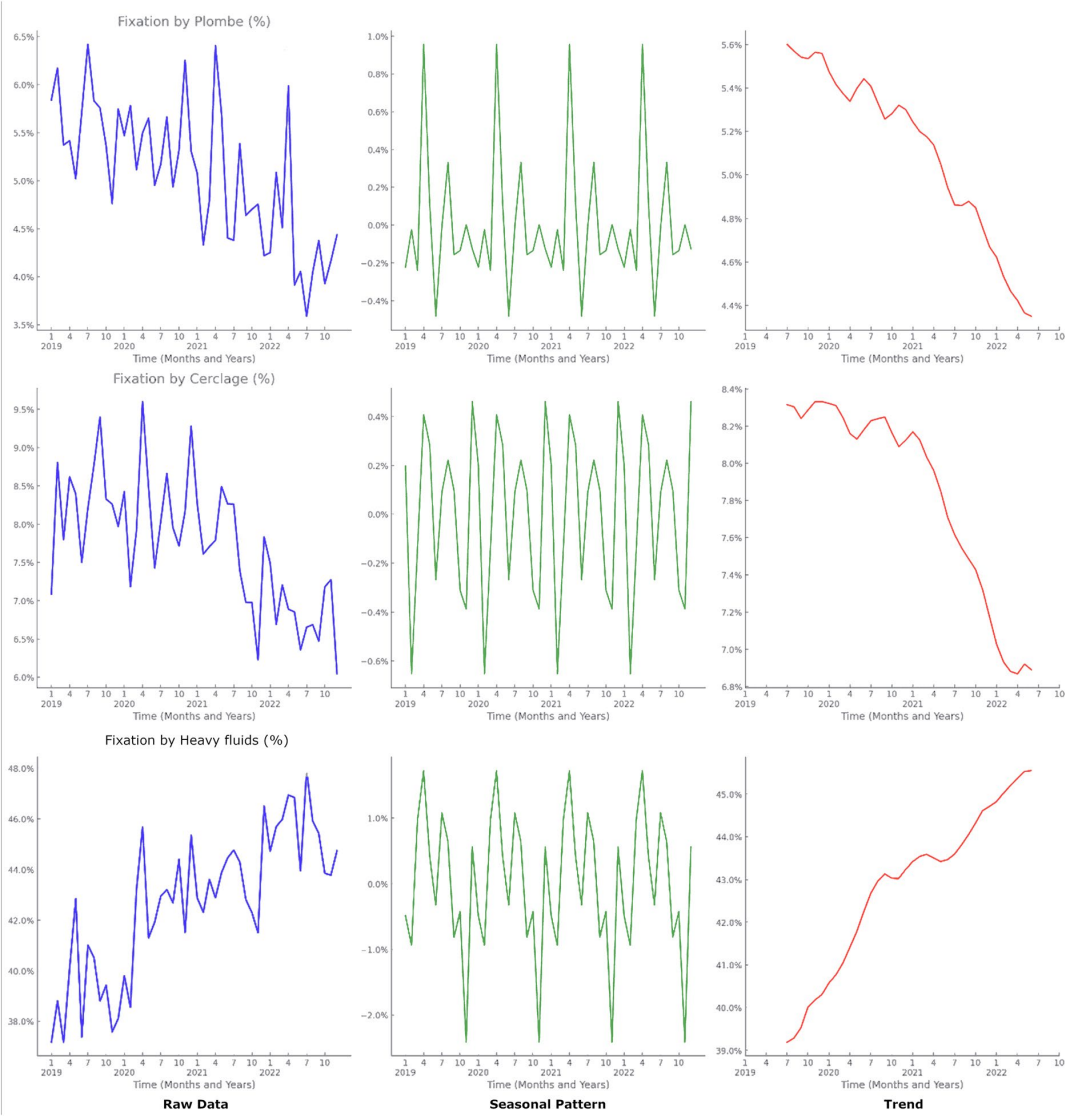
Seasonality of rhegmatogenous retinal detachment surgery type in percentage: Line 1 by Plombe (scleral buckle), Line 2 by Cerclage (encircling band) and line 3 by Heavy fluid

Analysis of surgical methods revealed shifts in utilization patterns post-pandemic. The use of fixation through permanent scleral buckling and fixation by cerclage (encircling band) decreased slightly, while the utilization of other retinal fixation operations involving heavy fluids increased significantly from 39.3 (SD ± 1.7) pre-pandemic to 43.1% (SD ± 1.4) post-pandemic (*p* < 0.001). This increase was particularly evident in March and April (Fig. 3), suggesting a trend toward more definitive surgical interventions during the pandemic.

Reoperation rates for RRD showed a slight, non-significant increase from 12.1 pre-pandemic to 12.9% post-pandemic (*p* = 0.093). For TRD, reoperation rates increased marginally from 13.4 to 14.1% post-pandemic. Mean admission time for RRD cases decreased from 3.33 days (SD ± 0.06) pre-pandemic to 3.19 days (SD ± 0.09) post-pandemic (*p* < 0.001), indicating that hospitals may have implemented strategies to shorten inpatient stays during the pandemic (Supplementary Fig. 3).

### Hospital characteristics

The proportion of RRD cases managed in larger hospitals with 1,000 beds or more decreased from 55.0 (SD ± 1.0) pre-pandemic to 51.4% (SD ± 1.3) post-pandemic (*p* < 0.001). Admissions to nonprofit hospitals slightly increased from 13.1 to 15.1% (*p* < 0.001), while public hospitals remained the primary providers without significant change. These shifts may reflect changes in resource allocation and capacity challenges faced by larger institutions during the pandemic.

### Correlation with reoperation rates

Spearman correlation analysis demonstrated a statistically significant positive correlation between monthly COVID-19 deaths and reoperation rates in RRD cases (correlation coefficient = 0.36, *p* = 0.011). Mean admission time was negatively correlated with reoperation rates (correlation coefficient = − 0.36, *p* = 0.011), suggesting that shorter hospital stays were associated with higher reoperation rates. Additionally, the percentage of cases undergoing fixation by cerclage was negatively correlated with reoperation rates (correlation coefficient = − 0.32, *p* = 0.026) (Supplementary Table 2).

Granger causality tests with a lag of four months indicated that decreases in RD admissions and reductions in admission duration during the pandemic could contribute to increased reoperation rates in subsequent months (*p* = 0.015 and *p* = 0.016, respectively). These findings suggest that pandemic-related disruptions may have had delayed effects on surgical outcomes.

## Discussion

This study provides a comprehensive analysis of the impact of the COVID-19 pandemic on RD cases in Germany from 2019 to 2022. Our findings reveal significant disruptions in RD incidence, seasonality patterns, surgical management, and hospital resource allocation, offering valuable insights into how a global health crisis can affect the management of time-sensitive ophthalmic conditions.

**Fig. 4:**
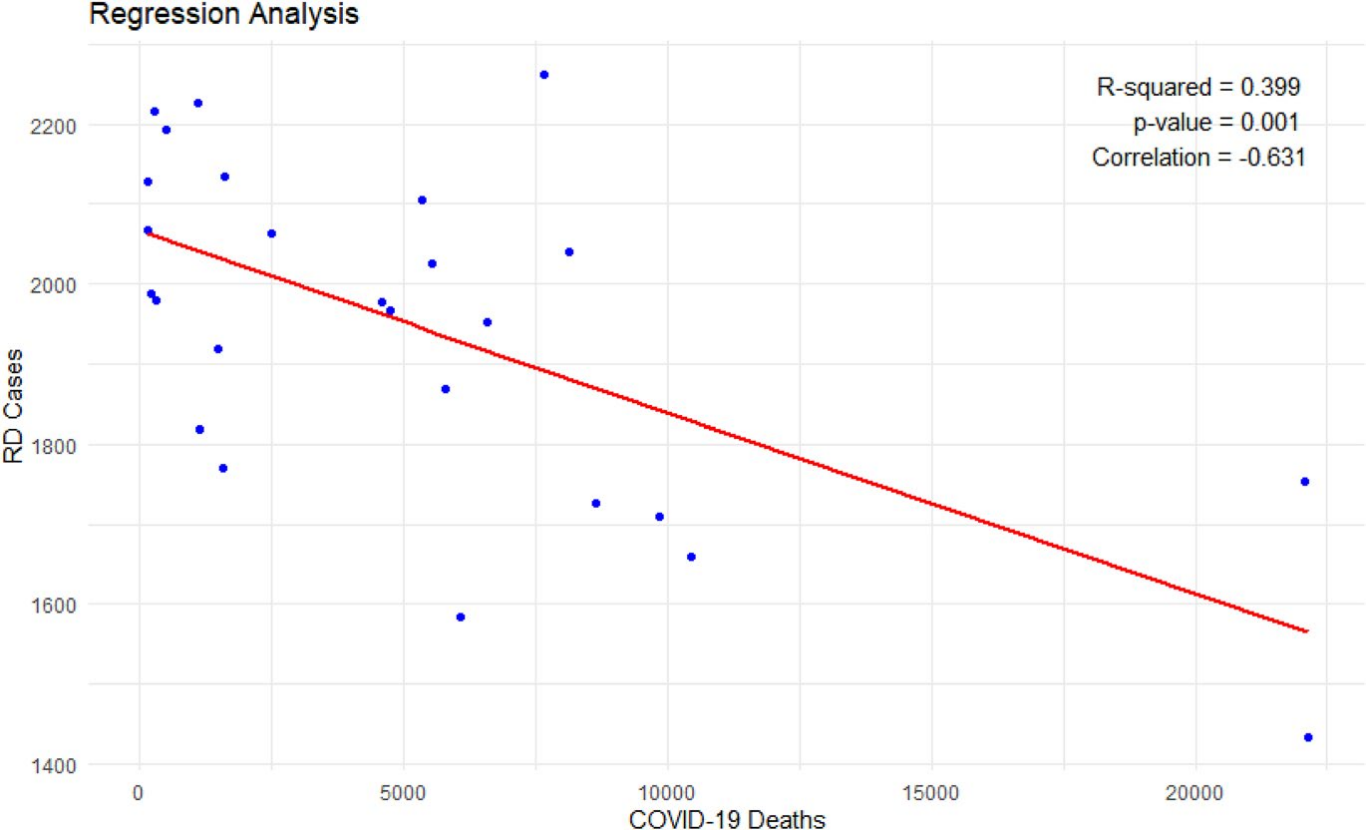
Regression analysis between Number of COVID 19 Deaths and RRD admissions

The observed decrease in RRD admissions during the pandemic aligns with reports from other regions, indicating a global trend of reduced ophthalmic presentations amid COVID-19 surges [13, 14]. The 8.5% decline in RRD cases post-pandemic onset suggests that patients may have deferred seeking care due to fear of infection, lockdown measures, or healthcare system strain. It can be also related to the decrease in daily activity. This is supported by the significant negative correlation between monthly RRD admissions and COVID-19 deaths (R^2^ = 0.40, *p* = 0.001), indicating that higher mortality rates, reflecting pandemic severity, were associated with fewer RRD admissions (Fig. 4). Similar patterns have been reported in other studies, where anxiety related to COVID-19 and access barriers led to delayed presentations of ocular emergencies [15, 16]. While it cannot be entirely excluded that a minority of patients underwent treatment in an ambulatory setting (e.g., in-office procedures), such cases are generally not reimbursed under the German healthcare system, which mandates hospital admission for RRD surgery. Therefore, it is unlikely that this accounts for the observed 8% of cases.

In contrast, TRD admissions experienced a smaller decline of 2.9%, suggesting that TRD cases were less sensitive to pandemic-related disruptions. TRD often develops gradually and is commonly associated with underlying conditions like diabetic retinopathy, allowing for more flexible scheduling compared to the urgent intervention required for RRD, especially when the macula is on [1, 17]. The differential impact on RRD and TRD emphasizes the need for tailored healthcare strategies during public health crises, prioritizing conditions with higher risks of rapid visual deterioration.

Seasonality patterns in RD admissions were disrupted during the pandemic. Traditionally, RRD cases peak during summer months, mainly July, which may be attributed to increased outdoor activities, physical trauma, and UV exposure altering the vitreous body structure [18]. Our data showed that this peak shifted to May in 2022, possibly reflecting changes in public behavior or delayed healthcare-seeking as restrictions eased. Understanding these seasonal trends is crucial for healthcare planning, as they significantly impact the demand for emergency ophthalmological services. These seasonal variations are crucial for healthcare planning, as they can significantly impact the demand for emergency ophthalmological services [19]. This notion as well as the overall decrease of RD surgery might indicate that some of the RD cases might be triggered by outdoor activity.

The pandemic also influenced surgical management of RRD. We observed a significant increase in the use of vitrectomy procedures involving heavy fluids, rising from 39.3 pre-pandemic to 43.1% post-pandemic (*p* < 0.001), and a slight decrease in traditional methods like scleral buckling (Fig. 3). This shift may be due to surgeons favoring more definitive procedures that potentially reduce the need for reoperations and follow-up visits, thereby minimizing patient exposure to healthcare settings during the pandemic [20].

Despite adaptations in surgical practices, reoperation rates for RRD increased slightly post-pandemic from 12.1 to 12.9%, though not statistically significant (*p* = 0.093). The positive correlation between reoperation rates and COVID-19 deaths (correlation coefficient = 0.36, *p* = 0.011) suggests that pandemic pressures coincided with changes in surgical outcomes. Shorter mean admission times post-pandemic (from 3.33 to 3.19 days, *p* < 0.001) were negatively correlated with reoperation rates (correlation coefficient = − 0.36, *p* = 0.011), indicating that efforts to reduce inpatient duration may have unintended consequences on patient recovery and the need for additional interventions. However, this declining trend in admission time was already observed since the implementation of DRG system [1].

The urgency of addressing RD is underscored by the sight-threatening nature of the condition, often necessitating prompt surgical intervention [13]. In contrast, TRD cases can afford more time for preoperative assessment and planning unless there is a combined rhegmatogenous component, which accelerates progression and necessitates earlier intervention. Surgical outcomes for RD have been a subject of investigation, with studies assessing the impact of the COVID-19 pandemic on these outcomes [14]. Additionally, the frequency of hospital admissions for surgically treated RD has shown a significant increase over the years [21].

Moreover, the association between COVID-19 impact and surgical urgency is highlighted by adaptations in surgical training and resident education. The pandemic necessitated changes in the training environment, including an increased reliance on simulation and virtual learning tools to maintain surgical skill development despite reduced patient volumes [22, 23]. This adaptation in training also reflects the broader operational shifts in ophthalmic practice, including how surgical procedures were prioritized and managed during the pandemic.

One notable observation from our study is the increasing trend in RD cases over time, mainly evident after the initial wave of the COVID-19 pandemic. A plausible explanation for this trend could be the hesitation among patients to seek medical care during the height of the pandemic. This hesitancy might have led to an accumulation of untreated RD cases, who then sought medical intervention as the healthcare system gradually adapted to the new normal, thereby contributing to the observed increase in RD cases [24, 25].

The increasing trend in RD cases post-COVID-19 and the shifts in healthcare resources and policies can be understood through several adaptations made by healthcare systems during the pandemic. Rigorous infection control measures, such as enhanced cleaning protocols and the use of personal protective equipment (PPE), were implemented widely across healthcare settings to mitigate transmission risks. These measures helped restore public confidence in seeking medical care, which might explain the observed increase in RD cases as patients felt safer accessing healthcare services [26–29]. Moreover, healthcare policies evolved rapidly in response to the pandemic. Changes included modifications to telemedicine regulations and the easing of restrictions on elective surgeries. These policy shifts made it easier for patients to access care for non-COVID conditions, including RD, as healthcare systems adapted to the ongoing pandemic conditions [30]. These combined factors—enhanced safety protocols, strategic resource management, and adaptive policy changes—played crucial roles in shaping patient access to RD treatment during and after the peak periods of the COVID-19 pandemic.

Hospital characteristics shifted during the pandemic, with a decrease in RRD cases managed by larger hospitals (from 55.0 to 51.4%, *p* < 0.001) and an increase in admissions to nonprofit hospitals (from 13.1 to 15.1%, *p* < 0.001). Larger institutions may have reallocated resources to COVID-19 care, reducing capacity for elective or semi-urgent surgeries [31]. These changes highlight the importance of flexible healthcare systems capable of redistributing workload across different hospital types during crises.

While our study offers valuable insights into the impact of the COVID-19 pandemic on retinal detachment cases in Germany, it has limitations that warrant consideration. Firstly, its retrospective design constrains the ability to establish causal relationships and control for confounding variables. Secondly, the use of a secondary data source, the DRG statistics database, introduces the potential for coding errors or omissions, and the accuracy of our findings is contingent upon the quality of the initial data entry. Additionally, the temporal scope, covering January 2019 to December 2022, may not capture long-term impacts of the pandemic. Our dataset also lacks clinical patient-level details such as initial presentation, comorbidities, lifestyle factors, and socioeconomic status, restricting a more nuanced understanding of RD incidence and treatment outcomes. Lastly, despite statistical adjustments, inherent biases such as reporting errors and seasonal variations could potentially confound the results.

As an observational study using administrative data, our findings are subject to potential confounding, and we caution that observed associations do not imply causation. Factors such as healthcare system strain, changes in health-seeking behavior, or simultaneous policy adaptations may have contributed to the observed trends. Unmeasured confounding variables, including simultaneous healthcare policy changes, variations in regional hospital capacity, or undocumented shifts in outpatient management, may influence both COVID-19 deaths and RD admissions.

Our study provides a comprehensive analysis of the impact of the COVID-19 pandemic on RD cases in Germany, filling a gap in existing literature. We observed a significant seasonality in RD incidence, which appeared to be disrupted by the pandemic. Furthermore, there was a negative correlation between COVID-19 deaths and RD-related admissions, suggesting a decline in RD surgeries during pandemic peaks, possibly due to healthcare disruptions or patient hesitancy. Interestingly, we noted an increasing trend in RD cases over time, potentially attributable to an accumulation of untreated cases who sought medical care as healthcare systems adapted. These findings have critical implications for healthcare policy and clinical practice, particularly in preparing for future public health crises. Further research is needed to corroborate these findings and explore the long-term consequences of the pandemic on RD and ophthalmic care more broadly.

## Supplementary Information

Below is the link to the electronic supplementary material.

Supplementary file1 (DOCX 60 KB)

## Data Availability

Datasets related to this article can be found at https://www.g-drg.de/inek-datenportal, an open-source online data repository hosted by the Institute for the Remuneration System in Hospitals.
