# Supplementary material for: COVID-19 impact on retinal detachment in Germany: seasonal peaks persist and shifts in surgical trends
Source: Int Ophthalmol. 2025 Sep 1;45(1):369. doi: 10.1007/s10792-025-03706-z (PMC12402007; doi:10.1007/s10792-025-03706-z)
Supplement: Supplementary file 1 — Supplementary file1 (DOCX 60 KB) [file 10792_2025_3706_MOESM1_ESM.docx]

Supplementary Tables:

Supplementary Table 1. Comparing the 12-month period before March 2020 to that after (including March month)

| Variable | |  | Mean (Pre-COVID) | SD (Pre-COVID) | Mean (Post-COVID) | SD (Post-COVID) | p-value (t-test) |
| --- | --- | --- | --- | --- | --- | --- | --- |
| N | |  | 23910 | | 21876 | |  |
| RD Cases | |  | 1992.5 | 194.35 | 1823.0 | 202.89 | 0.048 |
| Sex: Males (%) | |  | 64.6 | 1.3 | 64.3 | 0.9 | 0.586 |
| Mean Admission Time (days) | |  | 3.33 | 0.06 | 3.19 | 0.09 | 1.01×10^−4^ |
| Age: Late Work | |  | 52.6 | 1.2 | 52.5 | 0.01.7 | 0.880 |
| Age: Post Work | |  | 42.0 | 0.01.1 | 41.9 | 0.01.8 | 0.880 |
|  | Management (%) | | | | | | |
| 5-152.0: Fixation by indentation operations | |  | 5.6 | 0.4 | 5.3 | 0.5 | 0.140 |
| 5-152.2: Fixation by cerclage | |  | 8.2 | 0.6 | 8.3 | 0.7 | 0.932 |
| 5-154.4: Fixation by heavy fluids | |  | 39.3 | 1.7 | 43.1 | 1.4 | 4.08×10^−6^ |
| 5-983: Reoperation | |  | 12.1 | 1.2 | 12.9 | 1.2 | 0.093 |
|  | Hospital Size and type | | | | | | |
| 1000 beds and more (%) | |  | 55.0 | 1.0 | 51.4 | 1.3 | 1.23×10^−7^ |
| Non-profit (%) | |  | 13.1 | 0.7 | 15.1 | 1.0 | 5.41×10^−6^ |
| Public (%) | |  | 71.2 | 0.7 | 71.3 | 0.9 | 0.706 |

Supplementary Table 2. Examining the correlation, and Granger causality (at lag =4), between different variables and reoperation rates monthly

| Factor | Spearman Correlation | Spearman P-value | Granger Causality P-value |
| --- | --- | --- | --- |
| CoViD19 Deaths | 0.36 | **0.011** | **0.067** |
| RD Cases | -0.09 | 0.553 | **0.015** |
| Demographics | | | |
| Sex: Males | -0.10 | 0.502 | 0.624 |
| Age – Late-Work | 0.06 | 0.689 | 0.690 |
| Age – Post-Work | -0.07 | 0.620 | 0.406 |
| Mean Admission Time | -0.36 | **0.011** | **0.016** |
| Management | | | |
| Fixation by Plombe (%) | -0.21 | 0.158 | 0.102 |
| Fixation by Cerclage (%) | -0.32 | **0.026** | 0.241 |
| Other Retinal Fixation by Heavy Fluids (%) | 0.13 | 0.363 | 0.145 |
| Endotamponade (gas) (%) | 0.07 | 0.659 | 0.210 |
| ppV with Removal of Membranes + Other Gases (%) | -0.22 | 0.139 | 0.903 |
| Hospital Category | | | |
| Hospitals with 1000 beds and more (%) | -0.05 | 0.715 | 0.823 |
| Nonprofit Hospitals (%) | 0.22 | 0.140 | 0.223 |
| Public Hospitals (%) | 0.15 | 0.317 | 0.790 |

*ppV: Pars plana vitrectomy

Figures:


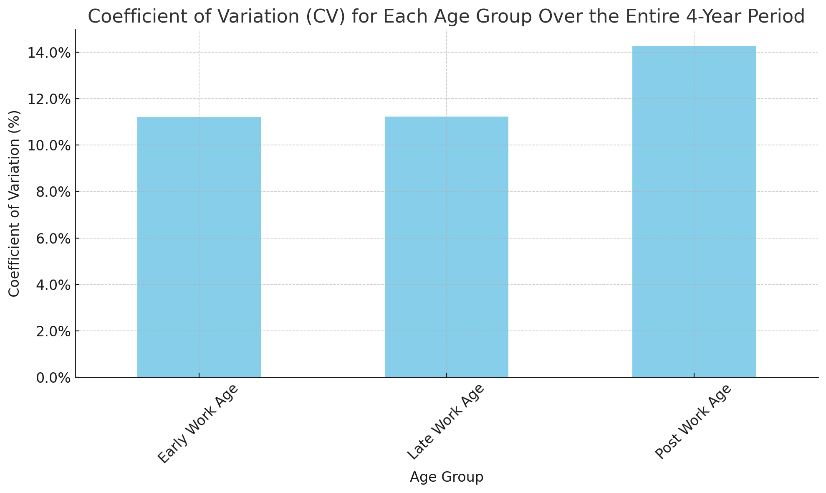
Supplementary figure 1. Re-Representation of figure 1, comparing number of cases in each month per year


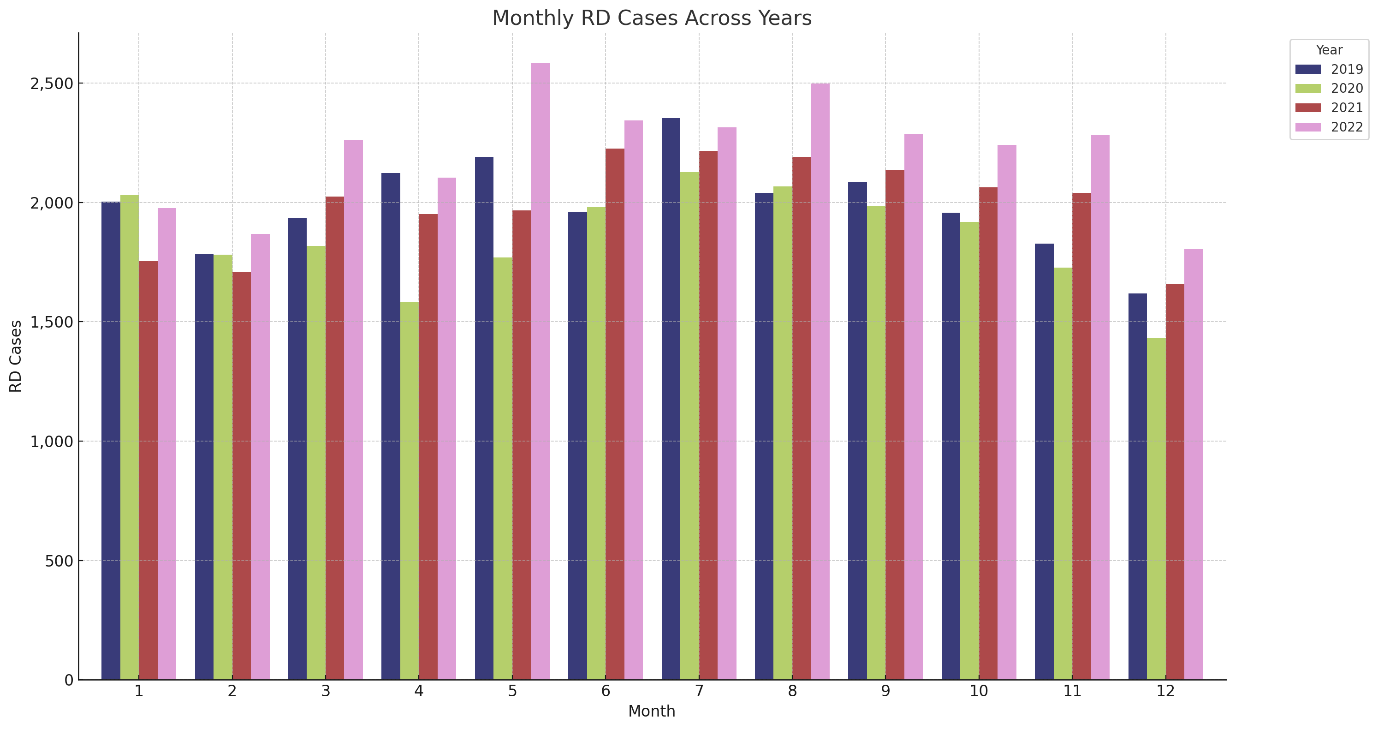


Supplementary Figure 2. The age groups affection by the lockdown. The coefficient of variation represents the changes influenced by Covid19


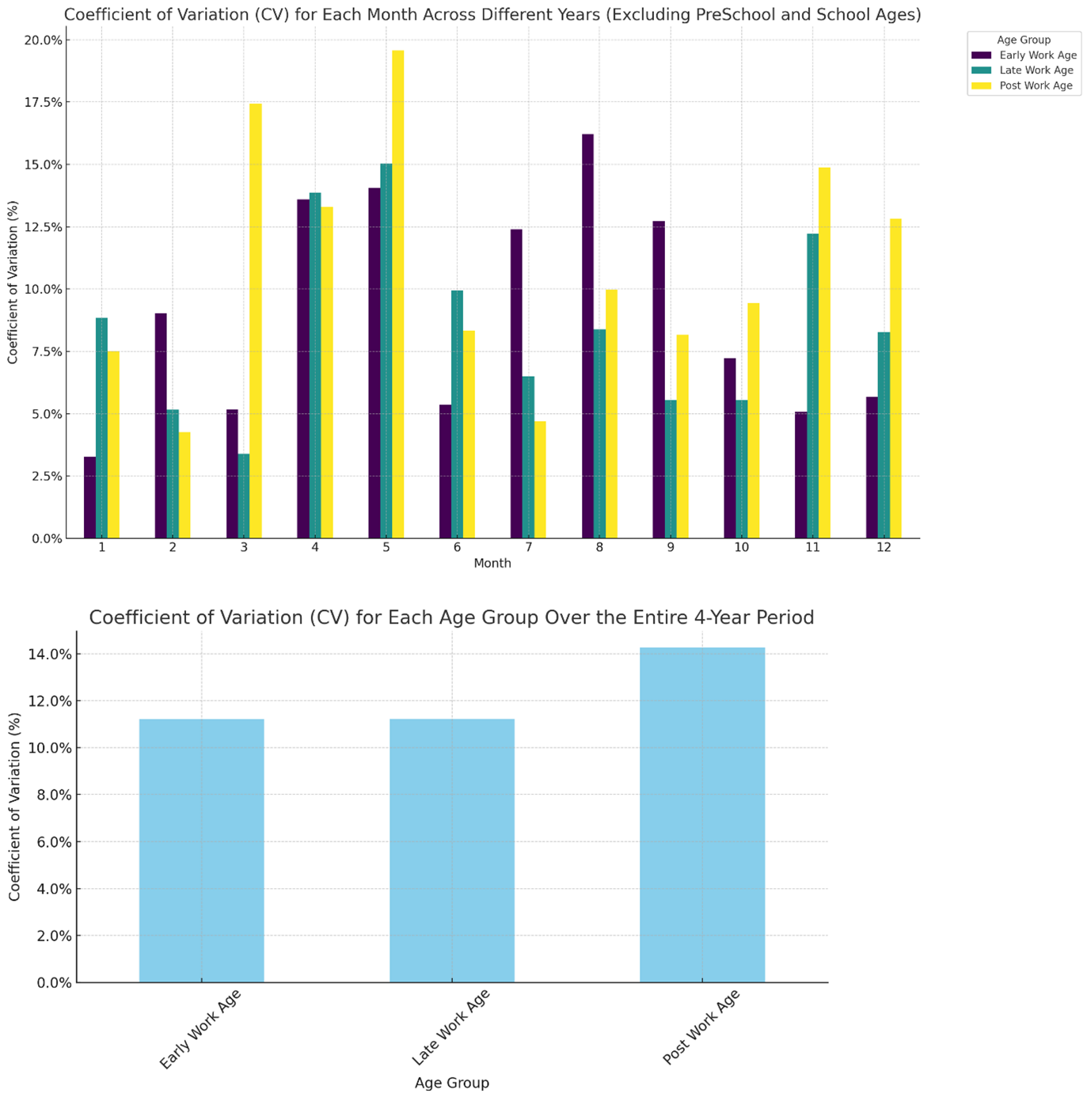


Supplementary Figure 3. Timeseries decomposition of reoperation rates


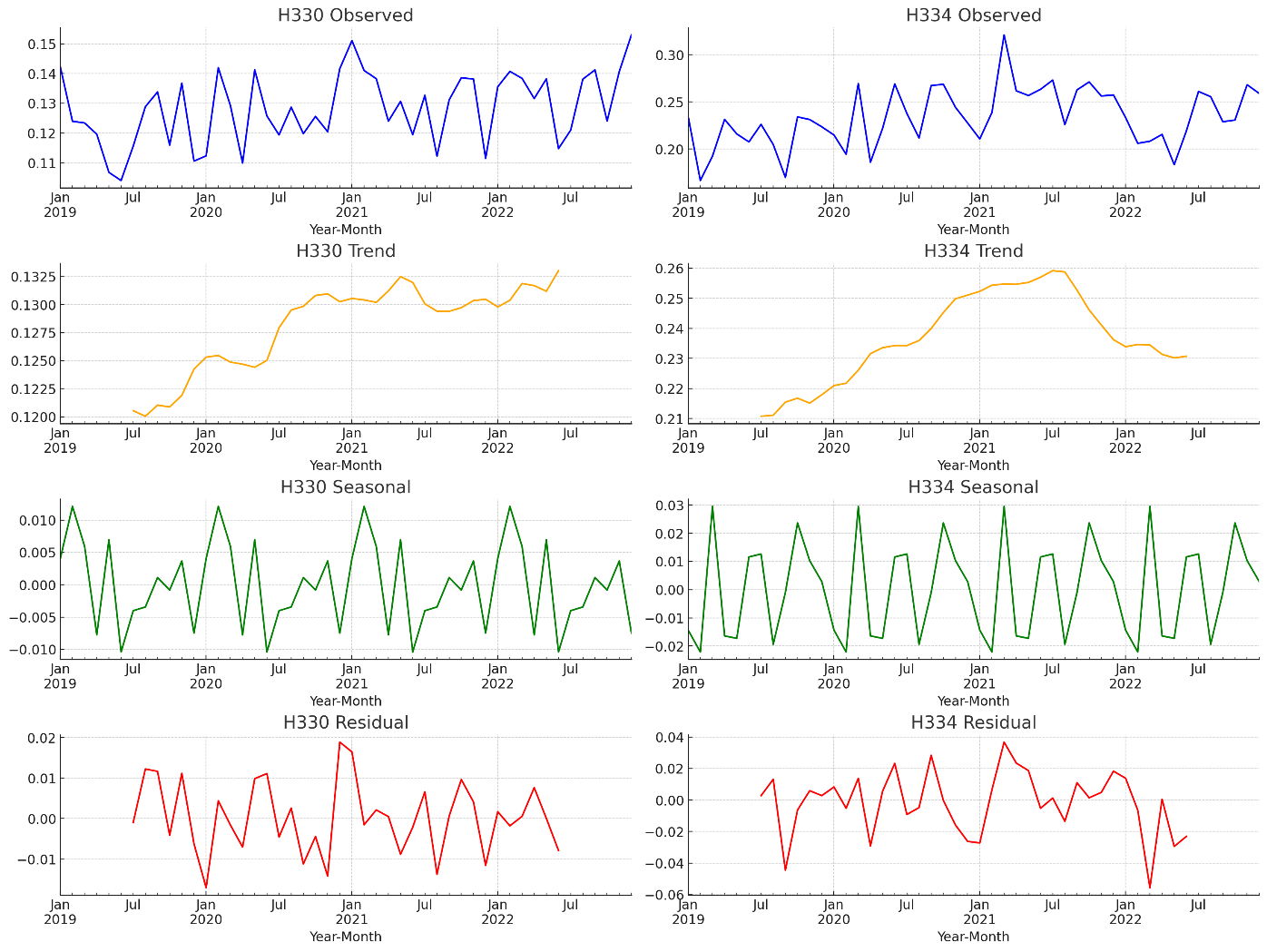


Supplementary Figure 4. Correlation between reoperation rates in both Rhegmatogenous and tractional retinal detachment.
